# Implementing stroke care in a lower-middle-income country: results and recommendations based on an implementation study within the Nepal Stroke Project

**DOI:** 10.3389/fneur.2023.1272076

**Published:** 2023-10-24

**Authors:** Christine Tunkl, Raju Paudel, Sunanjay Bajaj, Lekhjung Thapa, Patrick Tunkl, Avinash Chandra, Bhupendra Shah, Balgopal Karmacharya, Ashim Subedi, Pankaj Jalan, Pradesh Ghimire, Mahesh Raj Ghimire, Gampo Dorje, Nima Haji Begli, Jessica Golenia, Bikram Prasad Gajurel, Shirsho Shreyan, Nooma Sharma, Alexandra Krauss, Jeyaraj Pandian, Thomas Fischer, Jan van der Merwe, Wolfgang Wick, Werner Hacke, Christoph Gumbinger

**Affiliations:** ^1^Department of Neurology, University Hospital Heidelberg, Heidelberg, Germany; ^2^Department of Neurology, Grande International Hospital, Kathmandu, Nepal; ^3^Department of Neurology, University of Texas Health Science Center at Houston, McGovern Medical School, Houston, TX, United States; ^4^Department of Neurology, National Neuro Center, Kathmandu, Nepal; ^5^Department of Neurology, Annapurna Neurological Institute, Kathmandu, Nepal; ^6^Department of Neurology, B. P. Koirala Institute of Health Sciences, Dharan, Nepal; ^7^Department of Neurosurgery and Emergency Medicine, Manipal Teaching Hospital, Pokhara, Nepal; ^8^Department of Neurology, Norvic International Hospital, Kathmandu, Nepal; ^9^Chitwan Medical College Teaching Hospital, Bharatpur, Nepal; ^10^Tribhuvan University Teaching Hospital, Kathmandu, Nepal; ^11^WHO Country Office Nepal, Kathmandu, Nepal; ^12^Rajshahi Medical College, Rajshahi, Bangladesh; ^13^Christian Medical College, Ludhiana, India; ^14^Boehringer Ingelheim International GmbH, Ingelheim, Germany

**Keywords:** LMIC, stroke care advocacy, implementation, acute stroke care, quality, stroke, Nepal

## Abstract

**Background:**

Globally, the majority of strokes affect people residing in lower- and lower-middle-income countries (LMICs), but translating evidence-based knowledge into clinical practice in regions with limited healthcare resources remains challenging. As an LMIC in South Asia, stroke care has remained a healthcare problem previously unaddressed at a national scale in Nepal. The Nepal Stroke Project (NSP) aims to improve acute stroke care in the tertiary healthcare sector of Nepal. We hereby describe the methods applied and analyze the barriers and facilitators of the NSP after 18 months.

**Methods:**

The NSP follows a four-tier strategy: (1) quality improvement by training healthcare professionals in tertiary care centers; (2) implementation of in-hospital stroke surveillance and quality monitoring system; (3) raising public awareness of strokes; and (4) collaborating with political stakeholders to facilitate public funding for stroke care. We performed a qualitative, iterative analysis of observational data to analyze the output indicators and identify best practices.

**Results:**

Both offline and online initiatives were undertaken to address quality improvement and public awareness. More than 1,000 healthcare professionals across nine tertiary care hospitals attended 26 stroke-related workshops conducted by Nepalese and international stroke experts. Monthly webinars were organized, and chat groups were made for better networking and cross-institutional case sharing. Social media-based public awareness campaigns reached more than 3 million individuals. Moreover, live events and other mass media campaigns were instituted. For quality monitoring, the Registry of Stroke Care Quality (RES-Q) was introduced. Collaboration with stakeholders (both national and international) has been initiated.

**Discussion:**

We identified six actions that may support the development of tertiary care centers into essential stroke centers in a resource-limited setting. We believe that our experiences will contribute to the body of knowledge on translating evidence into practice in LMICs, although the impact of our results must be verified with process indicators of stroke care.

## Background

Stroke is the third leading cause of death and disability, with 89% of stroke-related deaths and disabilities affecting people residing in lower- and lower-middle-income countries (LMICs) (1). In LMICs, the burden of stroke is further exacerbated by limited access to healthcare and inadequate stroke care resources (2), with acute stroke care being provided to less than a third of all stroke patients (3).

In Nepal, an LMIC (4) in South Asia with a population of 30 million people, which is ranked 143 on the Human Development Index, stroke is the third leading cause of death (5). As of 2019, the governmental expenditure on health per capita is US$53 per year, and stroke care is primarily paid out-of-pocket (6). The lack of financial resources in the public health sector aggravates the lack of specialized healthcare personnel, and there is only approximately one neurologist per one million people (7). Compounding the difficulties is a low level of knowledge about stroke, leading to an under-use of health services and prehospital delay (8, 9). In response to these unmet needs, the Nepal Stroke Project (NSP) was initiated in 2021 with the aim of improving access to acute stroke care in Nepal's provincial tertiary care sector. The project was set up in a healthcare setting where multidisciplinary stroke unit care, intravenous thrombolysis, and endovascular treatment were restricted to private tertiary care centers in Kathmandu (7, 10, 11), and the recanalization rate was far <1% (12). Globally, LMICs such as Nepal face the challenge of translating evidence-based stroke knowledge into clinical practice, but specific guidelines or recommendations tailored to the needs of these resource-limited health settings are not yet available. Our study therefore aimed to analyze the implementation outputs after the first implementation phase (18 months) of the NSP and identify barriers and facilitators of the project's approach. The results of this analysis may then provide a valuable blueprint for regions that are going through a comparable process of establishing structured stroke care.

## Methods

### Study design

We conducted a prospective study in Nepal to analyze the output indicators and identify barriers and facilitators in the implementation of acute stroke care in Nepal's tertiary care sector.

### Setting

The study was performed between 2021 and 2023 in nine tertiary care centers in six different provinces of Nepal as part of the NSP, a collaboration between the Nepal Stroke Association (NSA) and the University Hospital Heidelberg with support from the Hospital Partnerships program of the Deutsche Gesellschaft für Internationale Zusammenarbeit (GIZ) GmbH. Technical advice was given by Angels Initiative (13), Christian Medical College Ludhiana, and the World Stroke Organization (WSO).

### Participants

The criteria for including hospitals in the study encompassed the availability of structural requirements for essential stroke services as defined by the WSO Roadmap for Stroke Services (14) (e.g., CT scan facilities, 24/7 emergency departments, and intensive care units) and participation agreements with hospital boards. Initially, a strategic planning and hospital service assessment using the WSO roadmap was conducted, and the election of study sites from all possible sources was based on a reasonable geographic coverage of all regions, preferring government-led healthcare institutions that cater to an estimated annual caseload of ~500 stroke patients. Our target was to include one hospital in each of Nepal's seven provinces. All health professionals >18 years old associated with the included hospitals were considered study participants without any prespecified exclusion criteria.

The multifaceted, multi-level approach from hospital to national level recognizes that healthcare systems operate at multiple levels, and interventions address participants at different levels as described in Table 1.

**Table 1:** Target groups, roles, and expected impact.

**Level**	**Target group**	**Defined role**	**Expected outcome of their involvement**
Project level	National Stroke Society, University Hospital Heidelberg	Provide leadership and stroke education, coordinate training, provide standardized guidelines, foster research, and advocate stroke care	Advocacy, capacity building, and sustainability
Hospital level	Physicians, Nurses	Participate in training, provide training, apply required knowledge, provide quality stroke care, monitor data, and engage in research	Quality stroke care, expertise, and role models
	Hospital boards	Support reorganization of services and foster education	Quality stroke care and education
	Patients	Recipients of improved stroke care	Improved outcome of stroke
	Patients' attendants	Learning about the severity and signs of stroke	Improved reporting rate to seek hospital care decreased door-to-needle time
Population level	Social media users; and the general population	Recipients of awareness campaigns	Increased stroke awareness
Governmental level	WHO Country Office Nepal, Ministry of Health and Population, local political leaders	Compiling and maintaining a national stroke registry and multi-level cross-organizational collaboration	Nationally ratified guidelines, allocation of human, and financial resources toward stroke care and education

### Description of intervention

**Figure 1:**
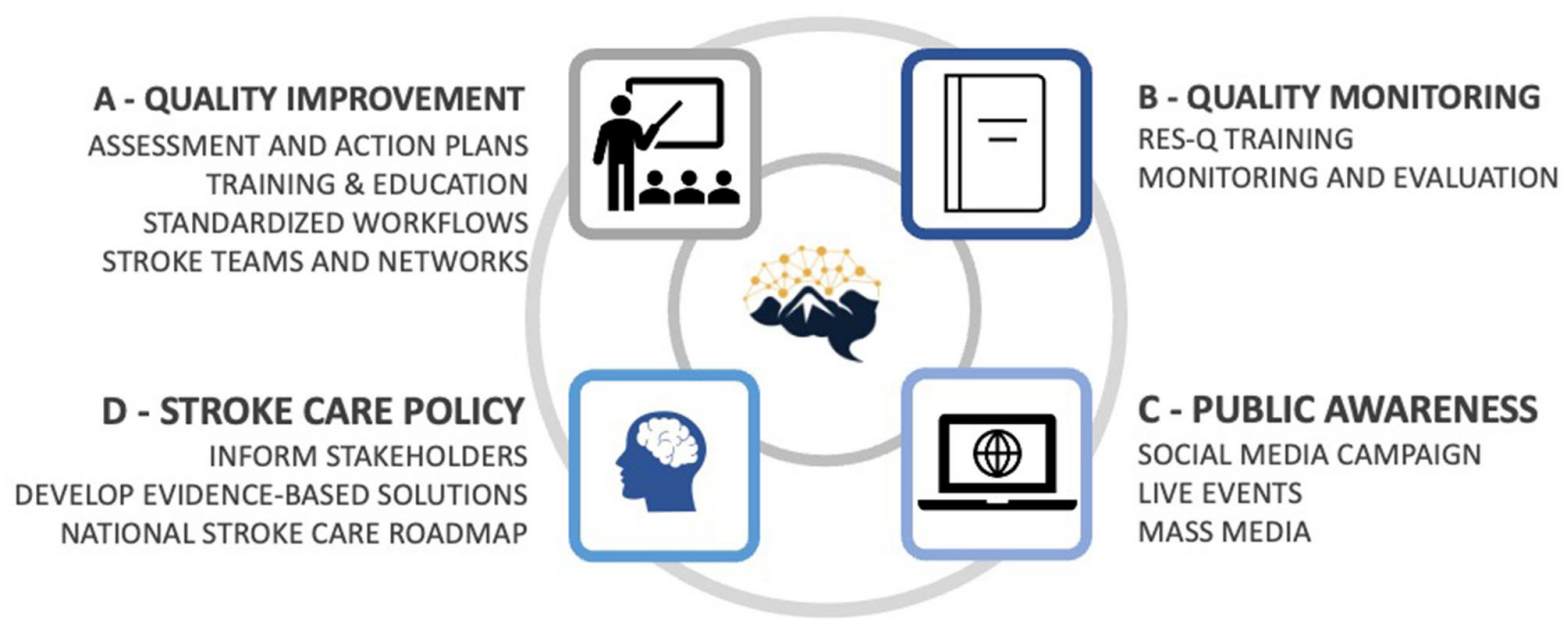
Strategies and key activities of the Nepal Stroke Project.

The project aimed to improve access to and quality of stroke care in Nepal's tertiary care sector, focusing on the acute in-hospital phases of care. The intervention combined four pillars (Figure 1):

Pillar A: Quality improvement—establishing stroke-ready hospitals in each province as defined by “essential stroke care” according to the WSO Roadmap (14).
Pillar B: Quality monitoring—implementing quality monitoring with the Registry of Stroke Care Quality (RES-Q) (15).
Pillar C: Public awareness—increasing public awareness of strokes.
Pillar D: Stroke care advocacy—providing evidence-based concepts of stroke care.

### Theoretical framework

The NSP is guided by the principles of community-based participatory research and empowers local health workers to provide culturally appropriate and responsive stroke care. The aim is to establish a self-sustaining network of advocates to build capacity at the grassroots level while simultaneously working with national-level policymakers to create an enabling environment for affordable stroke care.

**Figure 2:**
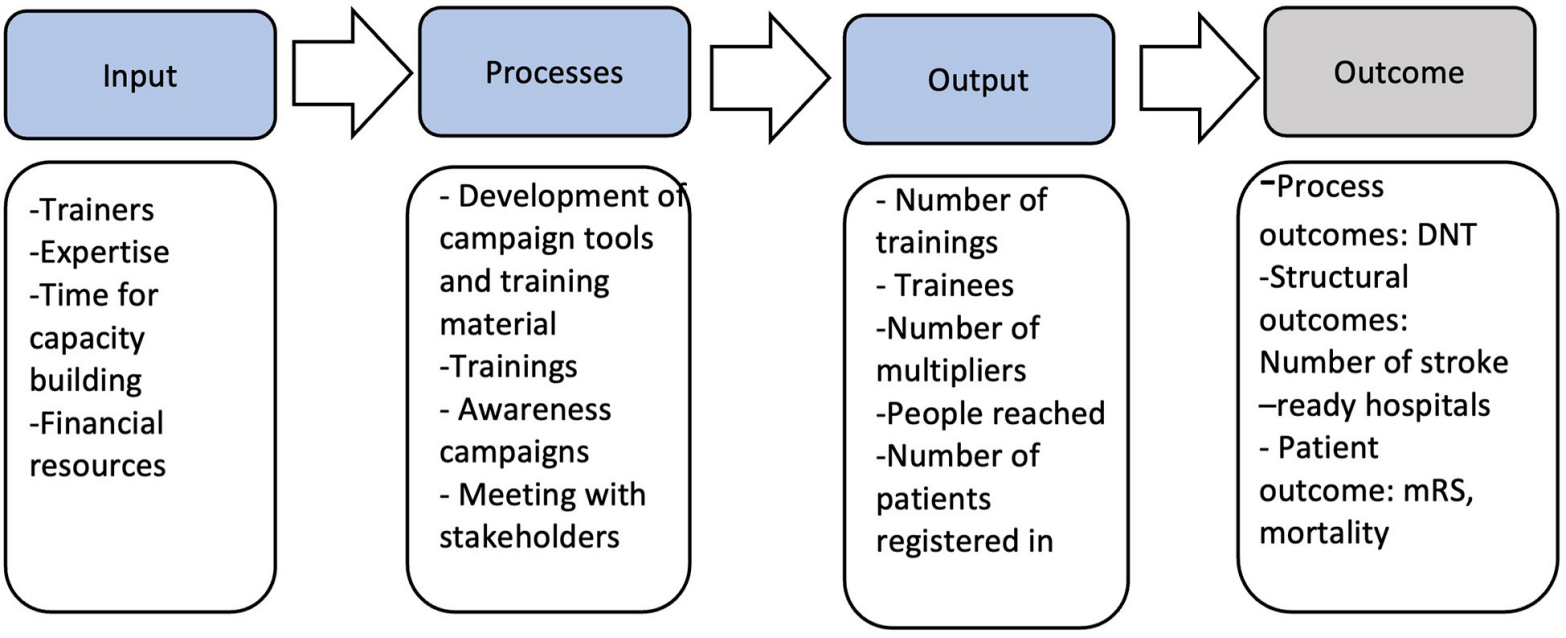
Logic model of project. DNT, door-to-needle time; mRS, modified Rankin Scale; RES-Q, Registry of Stroke Care Quality.

A logic model was developed to guide program planning, implementation, and evaluation (Figure 2) to ensure the project's alignment with the desired outcome. In clinical practice, a multifaceted approach that includes training programs for healthcare providers, stroke care advocacy, and stroke awareness campaigns has been devised.

### Data collection

Data collection adopted an iterative approach, extending from August 2021 to January 2023, and encompassed the utilization of the “World Stroke Organization Global Stroke Guidelines and Action Plans” and notes from on-site visits in all included hospitals, meeting records from all activities, project documents, and correspondences.

### Data analysis

Data relevant to quality improvement, quality monitoring, and stroke care advocacy were extracted from multiple sources (field notes, training records, and meeting notes). The data extraction process involved systematic collection to ensure comprehensiveness. The extracted data were then categorized into meaningful categories, such as:

- Level of Care: Data related to the level of care provided, in alignment with the WSO roadmap, would be included in this category. This might involve adherence to best practices and guidelines in stroke care.
- Quality Improvement Initiatives: This category includes data related to the hospitals' efforts to enhance the quality of stroke care. It may encompass records of training programs, quality improvement projects, and compliance with stroke care protocols within the hospital's clinical processes, including the frequency and consistency of their use.
- Training and Personnel: This category focuses on data pertaining to the number of training sessions conducted and the number of healthcare personnel trained in stroke care. This may also include data on the qualifications and experience of the trained staff.
- Continuous Quality Monitoring: This category involves data on the hospitals' practices for ongoing quality monitoring and evaluation of stroke care services. This may encompass performance metrics, audit reports, and feedback mechanisms.

#### Dependent variables

The study's dependent variables of interest are output measures that reflect the effectiveness of quality improvement efforts in stroke care (e.g., number of training sessions, number of trained healthcare personnel, and the extent to which hospitals engage in ongoing quality monitoring of their stroke care services).

#### Independent variables

The independent variables in this research are the characteristics of the hospitals themselves (e.g., hospital size and teaching status), ownership (public, private, and non-profit), and resource availability (e.g., staff and technology).

#### Identifying patterns

To identify patterns, a comprehensive thematic analysis was conducted. We aimed to discern any significant correlations, trends, or associations between hospital characteristics and the variables related to quality improvement and stroke care.

### Ethical clearance

This study was approved by the Nepal Health Research Council (Registration number 214/2021 P).

## Results

### Participants

**Figure 3:**
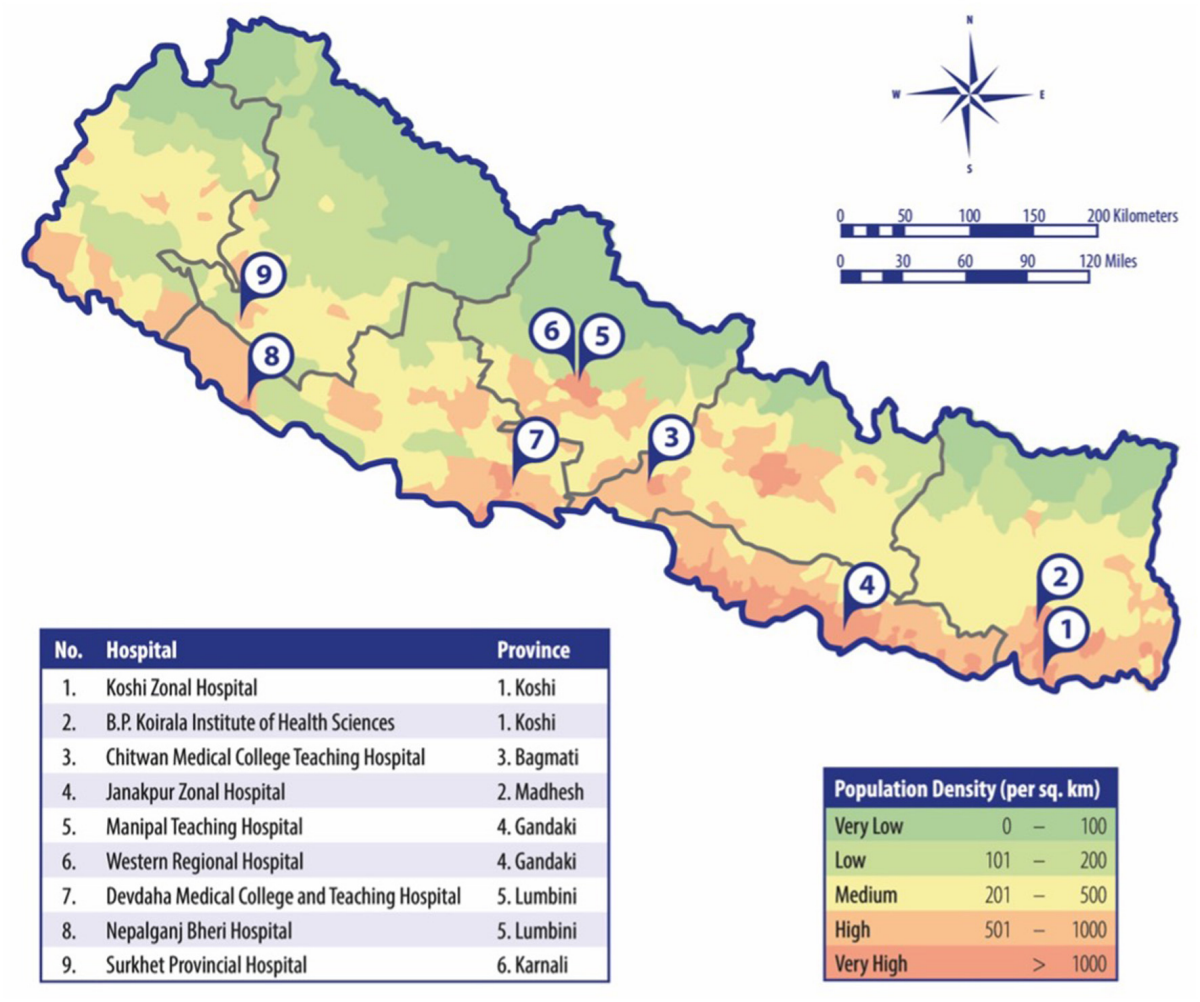
Geographical map of included hospitals.

Overall, 26 hospitals in Nepal were identified as possible study sites. Based on the geographic distribution, the catchment area, the ownership status, and the consent of the hospital boards, nine hospitals were included in the study (see Figure 3), covering six of the seven provinces of Nepal. Six hospitals were government-led centers, and three were semi-private teaching hospitals. None of the hospitals had a department of neurology or a dedicated stroke unit. The estimated number of stroke patients (ischemic and hemorrhagic) was on average between 50 and 100 per month.

### Output measures

#### Pillar A: quality improvement

Enhancing stroke treatment demands the widespread availability of hospitals equipped with essential tools and skilled personnel. Consequently, it became imperative to establish at least one tertiary care center in each major city of Nepal as a dedicated stroke-ready hospital. The criteria for qualifying as an Essential Stroke Center, as outlined by the WSO Roadmap for Stroke Services (14), serve as the benchmark for defining a stroke-ready hospital in this context.

In the initial phase, a strategic planning and hospital service assessment using the WSO Roadmap for Stroke Services was conducted (14). Based on this, nine hospitals were categorized as having minimal stroke services, and specific action plans were formulated to guide these hospitals in their journey toward becoming essential stroke centers in line with the WSO Roadmap. In response to the lack of clear responsibility for stroke care, the establishment of dedicated stroke teams, helmed by a stroke team leader functioning as a beacon of guidance, was identified as a priority. Stroke teams were built in five hospitals, and we repeatedly assessed the progress with stroke team leaders and defined the next steps in implementation (Table 2).

**Table 2:** Strategies, activities, and outputs within 18 months.

**Strategy**	**Activity**	**Output**
Quality improvement	Assessment	Assessment of resources and capacities in six tertiary care centers
		Targets and defined action lists in five tertiary care centers
	Training and education	9 physicians “trained as trainers”; 5 CME; 15 on-site 2-day workshops in 6 hospitals: 1,000 participants in total
		Monthly webinars: 45 participants on average; 10 newsletters with the basics of stroke sent to 100 nurses
	Treatment optimization	Flowcharts, pocket cards, practical guidelines, training manual, and National Stroke Guideline created
	Networking and team building	Designated stroke teams in four tertiary care centers
		Eight chat groups (hospital-specific; nurses; and physicians) with regular exchange among 100 health professionals
Quality monitoring	Introduction of a tool	Nine hospitals received an introduction and access to RES-Q
Public awareness	Social media campaign	91 posts (77 organic and 14 paid advertisements) on four different channels reached 2.5 million social media users and 250,000 engagements
	Live events	Stroke awareness camps in three different cities with >1,000 visitors
	Ambient/print media	10,000 flyers distributed, digital billboards displayed, and banners displayed in 25 restaurants
Stroke care advocacy	Stakeholder engagement	Taskforce with WHO and Ministry of Health; five meetings held; local political and religious leaders involved in activities
	National Stroke Road Map	In preparation

Ten stroke team leaders were selected to participate in the Angels Initiative “Train-the-Trainer Workshop,” (13) which equipped them with the skills necessary to act as trainers and local stroke advocates. More than 1,000 health professionals (physicians and nurses) participated in five medical education events (CME) and 15 interactive workshops covering basic knowledge about acute stroke care. Four special nurse workshops and monthly educational newsletters were provided to over 100 nurses. Online tools such as monthly webinars and access to educational resources from the World Stroke Academy and Angels Initiative were encouraged. Practical guidance in the form of posters, flowcharts, process-centered checklists, and pocket cards was provided. These resources were specifically tailored to suit the local conditions and facilitate the implementation of standardized procedures. Chat groups (hospital-specific, profession-specific, and thematic) served as platforms for case discussions, the exchange of knowledge and experiences, and training activities.

A 2-day stroke symposium was organized, bringing together 70 members of stroke teams from all participating hospitals and providing a valuable opportunity for collaboration and sharing of insights (Table 2).

#### Pillar B: quality monitoring

All participating hospitals' stroke teams received hands-on training in RES-Q (15). Additionally, printed checklists containing all relevant patient information were distributed to facilitate data entry. Seven out of nine study hospitals registered in RES-Q, of which only two hospitals entered the patients' data continuously. Evaluation of stroke care quality at the hospital level was not started due to a lack of reliable data.

#### Pillar C: public awareness

Both offline and online initiatives were undertaken to address quality improvement and public awareness. A social media-based stroke awareness campaign reached 3 million individual social media users in Nepal with organic traffic and paid advertisements on four social media platforms (Instagram, Facebook, Twitter, and TikTok) (16). Videos in the Nepali language were created and displayed on digital billboards in five public places for a total of 30 days. Health camps were held in three major cities of Nepal (Kathmandu, Janakpur, and Dharan) with distribution of information materials, lectures, and counseling on vascular risk factors. A walkathon was organized in Kathmandu. Through a strategic partnership, 10,000 flyers were distributed with food deliveries, and printed banners were placed in 25 partner restaurants (Table 2).

#### Pillar D: stroke care advocacy

The project focused on effectuating governmental backing for stroke management by integrating stroke care into the curriculum and reimbursement of expenses associated with stroke care. In 2022, a stroke care task force was established between the project partners and the World Health Organization (WHO) Country Office Nepal, the Ministry of Health and Population (MOHP), and the Department of Health Services (DOHS) with regular meetings, giving an opportunity to discuss current gaps in stroke care and emphasizing priorities for improvement. A national stroke protocol and the accreditation of training manuals represent the first steps, while key components of a comprehensive National Road Map for Stroke Care are presently in progress. A systematic literature review on the status of stroke care was performed in the absence of a national stroke registry (12).

### Analysis of facilitators of and barriers to stroke care on a hospital level

Within 18 months, three tertiary care centers have progressed from minimal to essential stroke services. It must be noted that these centers are all teaching hospitals, indicating that larger, academic hospitals may be more conducive to such quick improvements in stroke care services. By comparing the steps undertaken in these hospitals in comparison to the other partner hospitals, facilitators of and barriers to stroke care were identified.

**Figure 4:**
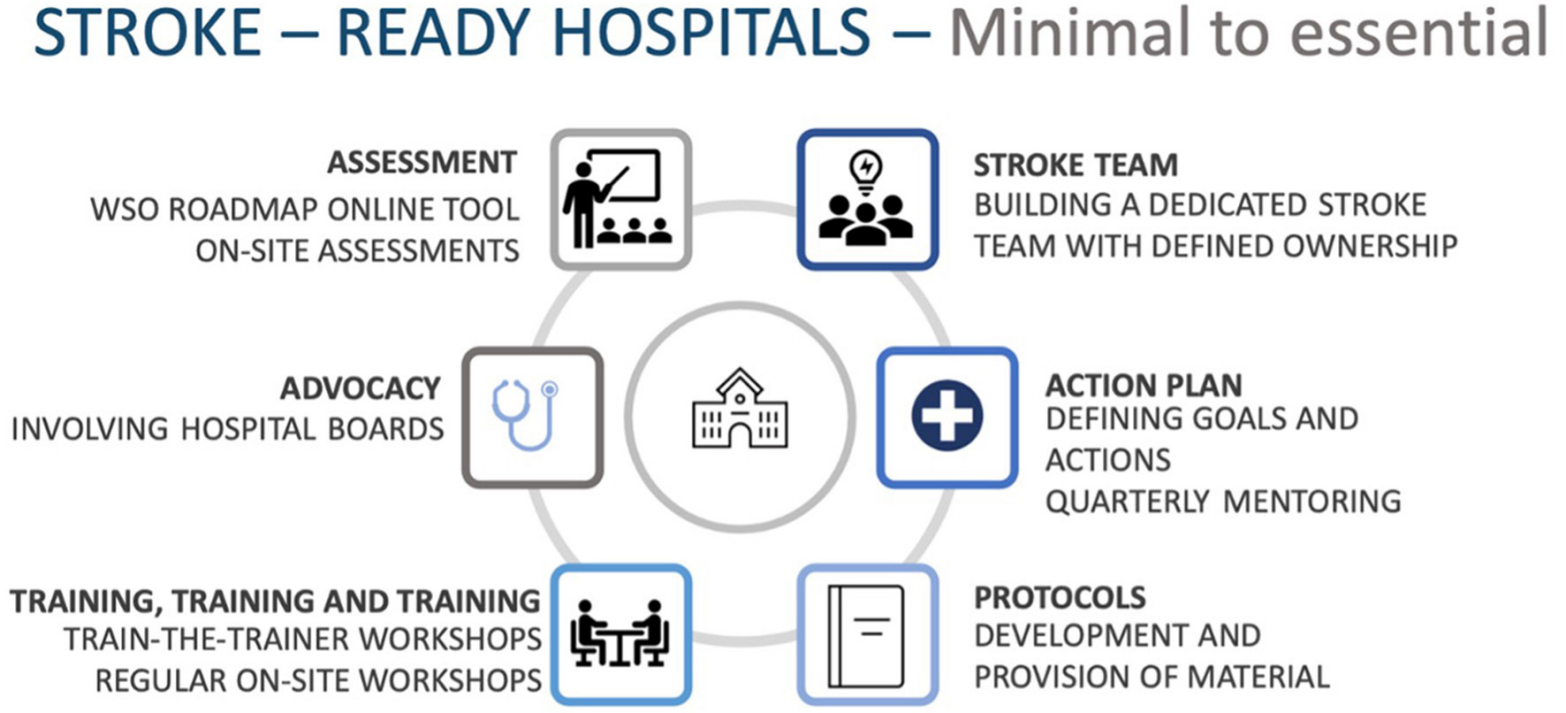
Identified facilitating actions in becoming a stroke-ready hospital.

One of the major determinants of the successful transition of these three centers from minimal to essential stroke services was the establishment of a core team of leaders, who took on the responsibility of organizing a structured stroke team at their institutions. This core team established an interdisciplinary collaboration between ED physicians, neurologists, neurosurgeons, nurses, and other ancillary staff to formulate ideal workflows, personnel requirements, and their respective goals. Teams organized continuous education events and were empowered to become trainers themselves. In the absence of neurologists in any of the hospitals, we leveraged physicians, internal medical professionals, and neurosurgeons to become stroke leaders. These core teams also had constant contact with their hospital administration and members at other institutions, such as University Hospital Heidelberg. In combination, we identified six facilitators for enhancing stroke care services on a hospital level (Figure 4).

In contrast, a disadvantageous factor at institutions that were unable to grow to essential care was a rapid turnover of personnel, which disrupted the development of stable core leadership groups. Moreover, insufficient or faulty technical equipment, particularly beds and imaging equipment, was severely detrimental to the efforts at improving stroke care.

Furthermore, the implementation of quality monitoring with RES-Q was unsuccessful, and our qualitative analysis indicates that this is to be attributed to the use of a tool that was selected only based on experiences from other countries but could not be easily integrated into the clinical routine and workflow of Nepal due to a lack of documentation systems and time constraints. This emphasizes the need for monitoring tools adapted to the needs of the Nepalese healthcare system.

## Discussion

The NSP represents a milestone in the advancement of stroke care in Nepal, and we hereby present the strategies, outputs, and identified barriers and facilitators of a multifaceted approach applied to enhance the quality of acute stroke care in an LMIC.

### Evaluation of project outputs

Our analysis indicates that the methodology employed by the NSP represents a promising and feasible approach for improving access to quality stroke care in regions where stroke care infrastructure is being implemented *de novo*. This accomplishment is reflected in the successful deployment of all activities of the multifaceted approach, culminating in the active engagement of six tertiary care centers in the program (as enumerated in Table 3), 26 educational events attended by over 1,000 participants, and a public awareness campaign that has reached nearly 3 million people throughout Nepal (Table 3). However, while the precise service outcome of the project after 18 months may not yet be quantifiable, the development of a dynamic stroke care movement that has influenced health personnel, social media users, health authorities, and political leaders is a vital accomplishment that has helped to lay the groundwork for a task force comprising political leaders who are likely to engender sustainable change at the system level of stroke care delivery. We identified four pillars that may support the successful implementation of stroke care in an LMIC, which are quality improvement, quality monitoring, public awareness, and stroke care advocacy.

**Table 3:** Recommendations.

**Step 1: Assessment and planning**
1.1. Do an assessment of stroke care in the country and define gaps in care using the WSO Roadmap.
1.2. Define a nationally owned roadmap.
1.3. Identify existing hospitals in each region with the potential to become stroke-ready hospitals.
1.4. Ensure the commitment of hospital boards and medical teams to actively participate in the program.
1.5. Clearly define your overall goal and strategies, and define the accountabilities for implementing the strategies.
**Step 2: Establishing stroke-ready hospitals**
2.1. Choose one aspect of stroke care to start with (e.g., hyperacute care).
2.2. Visit each participating hospital and assess the existing structures and facilities (e.g., process of care, diagnostic resources, protocols, and educational level of staff).
2.3. Build a stroke team in each hospital (physicians, nurses, and therapists) that has full ownership of implementation.
2.4. Define individual targets, milestones, and key actions to achieve these milestones.
2.5. Conduct training, training, training! Provide face-to-face and online educational tools.
2.6. Empower local health workers to become trainers themselves.
2.7. Develop and implement practical stroke care protocols.
2.8. Build a network of stroke teams and engage peer support.
**Step 3: Quality monitoring**
3.1. Do focus groups with local health workers to identify acceptable and suitable tools and methods for continuous quality monitoring.
3.2. Introduce the stroke registry.
3.3. Define a designated data manager and team in each hospital.
3.4. Analyze the data and identify areas for improvement.
**Step 4: Public awareness**
4.1. Address the general population through different modalities (social media, health camps, and flyers).
4.2. Involve marketing/communication experts and use available resources (WSD Campaign).
4.3. Use public awareness to support stroke care advocacy.
**Step 5: Stroke care advocacy**
5.1. Form a task force with relevant key stakeholders (political institutions, NGOs).
5.2. Regularly present progress to the task force and demonstrate to them opportunities in stroke care.
5.3. Create a National Stroke Care RoadMap to be agreed on by all stakeholders.
5.4. Use your local data to increase support for stroke care in the National Health Strategy (e.g., for funding, public awareness campaigns, and education).

### Barriers and facilitators in the implementation of acute stroke care

Our study revealed the vital role of a dedicated stroke team and a local stroke champion in driving transformative processes for quality improvement. Clear goals tailored to each hospital, supported by the WSO Roadmap, facilitated successful implementation. Simplifying stroke care and increasing accessibility for non-specialist providers had a positive impact. However, we initially underestimated the challenges in the Nepalese healthcare system, such as high patient volumes and staff turnover. Resources were spread thin across hospitals, leading to some facilities being overwhelmed. Hospital-level stroke care requires a multifaceted approach with committed teams, realistic goals, simplicity, repetition, and effective advocacy (Figure 4). A national road map would be helpful in supporting the hospital's strategies and teams.

In critical evaluation of our quality monitoring pillar, we found that the project initially did not adequately consider the obstacles associated with quality monitoring (QM) in clinical routine. While RES-Q is a widely used tool (15), its acceptance in Nepal was found to be low. This highlights the importance of evaluating suitable methods and local capabilities when implementing QM in a particular region, rather than relying solely on the experiences of other countries. The situation we face is a self-perpetuating vicious cycle where the unaffordability of stroke treatment discourages physicians and the lack of data on disease burden and treatment hinders advocacy efforts to secure financing for stroke care. Yet, one of the main obstacles to gathering reliable data on disease burden and treatment is time constraints, a lack of perceived need, and insufficient support.

The dissemination strategy employed in our public awareness campaign demonstrated remarkable efficacy in its penetration of the general population, which can be partially attributed to the inclusion of marketing experts within our research team (16), leveraging the World Stroke Day Campaign, and adopting a multifaceted approach utilizing diverse modalities.

Although concrete steps are yet to be undertaken, a stroke movement has emerged, precipitating discussions among Nepalese health authorities. Facilitating this process was the early integration of the WHO as a pivotal stakeholder in the country, thereby fostering collaborative engagement in the project.

### Comparison with previous studies

Our approach aligns with previous initiatives undertaken by upper-middle-income nations in Eastern Europe and Brazil, wherein a structured training program, international collaborations, and rigorous quality monitoring were identified as key factors in improving stroke care quality (17–20). India, the neighboring nation of Nepal in South Asia, has attained significant achievements in stroke care, exemplified by a surge in the number of stroke units led by physicians, the integration of acute stroke care in the public health system, and an extensive engagement in stroke-related research activities (21, 22).

It is noteworthy that the challenges encountered while aiming for a 5% rate of recanalization in Nepal were not reflected in many of the implementation strategies described in the regions, which are targeting an improvement of the thrombolysis rate from 5% to 10% (19, 20, 23).

Especially in light of recent research, which revealed the deficiency of stroke care in many African countries (3, 24), our experiences and lessons learned can therefore serve as a valuable blueprint for those regions. However, we emphasize that addressing healthcare challenges will encounter unique and diverse obstacles depending on the region; therefore, it is essential to carefully listen to local care providers and gain a comprehensive understanding of their respective contexts and needs rather than imposing standardized, one-size-fits-all approaches. Yet, it must be noted that our qualitative analysis does not allow us to draw conclusions about the impact of the project on patients' outcomes.

### Implications of the findings

Drawing on our extensive experiences, both positive and negative, in implementing stroke care in the resource-limited healthcare setting of Nepal, we have synthesized a set of best practices that are highly informed by our learnings (Table 3).

### Strengths and limitations

This study will significantly contribute to the body of knowledge regarding which strategies can be successful in a resource-limited healthcare setting with a recanalization rate far below 1% to improve stroke care at a system level. While our strategies are based on the experiences of other middle-income countries, our project represents a pioneering effort in transferring practices to a resource-restrained region where stroke care needs to be established *de novo*. However, it must be noted that this observational study does not allow for quantitative analysis of markers and predictors of stroke care improvement. No randomization of study participants was performed, and the highly subjective nature of evaluations as well as the varying levels of care provided by the tertiary care centers involved in this study limit the inter-rater and inter-method reliability. Moreover, the current analysis is limited in its scope, as the evaluation is based on interim output indicators. Future analysis, planned after a project runtime of 5 years, is required to draw conclusions regarding whether the strategies have a positive impact on process and structural indicators and, last but not least, on patient outcomes. Further analysis should also assess the perceived client-centered impact of the program (e.g., acceptance, satisfaction, and others). The exponential growth of the project from a small-scale practical implementation to nationwide dimensions, combined with the ongoing challenges in hospital-based data collection, further exacerbates the drawbacks of this observational study.

### Implication for future research

While the NSP's first phase, with a planned runtime of 3 years, focuses on acute stroke care at the tertiary care level, further activities along the continuum of stroke care, from primary prevention to rehabilitation, are necessary. As Nepal is one of the least urbanized countries in the world, telestroke care could be a promising way to serve remote regions, and primary health centers should be strengthened in the management of complications and stroke recurrence. Above all, we must not let the most important and obvious goal slip out of sight, namely, to establish at least one comprehensive stroke center in each of Nepal's provinces with affordable and subsidized stroke services. Future research needs to delve into the analysis of process outcome parameters to gain deeper insights into the effectiveness of interventions and strategies. Conducting randomized controlled trials can provide more robust evidence of the impact of interventions in stroke care, allowing for causal inferences. Expanding our research efforts to multinational studies can help identify global trends and best practices in stroke care, fostering cross-border collaboration and knowledge sharing.

## Conclusion

This study describes the experiences of the implementation of a multifaceted stroke program in the resource-limited healthcare setting of Nepal. The four strategies (quality improvement, quality monitoring, public awareness, and stroke care advocacy) created a stroke care movement in Nepal. On the hospital level, we identified six facilitating actions for becoming a stroke-ready hospital, which are assessment, stroke team formation, defining concrete actions, adopting protocols, continuous training, and advocacy at the hospital level. Our findings have the potential to contribute to the knowledge base on translating evidence into practice in LMICs.

## Data availability statement

The original contributions presented in the study are included in the article/Supplementary material, further inquiries can be directed to the corresponding author.

## Author contributions

CT: Conceptualization, Data curation, Formal analysis, Funding acquisition, Investigation, Methodology, Project administration, Visualization, Writing—original draft, Writing—review and editing. RP: Conceptualization, Data curation, Investigation, Project administration, Resources, Writing—review and editing. SB: Conceptualization, Formal analysis, Investigation, Methodology, Resources, Writing—original draft, Writing—review and editing. LT: Data curation, Investigation, Methodology, Project administration, Resources, Writing—review and editing. PT: Conceptualization, Data curation, Project administration, Software, Visualization, Writing—original draft. AC: Data curation, Investigation, Writing—review and editing. BS: Data curation, Investigation, Methodology, Project administration, Writing—review and editing. BK: Data curation, Investigation, Writing—review and editing. AS: Data curation, Investigation, Writing—review and editing. PJ: Data curation, Investigation, Writing—review and editing. PG: Data curation, Investigation, Writing—review and editing. MG: Data curation, Investigation, Writing—review and editing. GD: Resources, Supervision, Writing—review and editing. NB: Investigation, Writing—review and editing. JG: Data curation, Investigation, Writing—review and editing. BG: Conceptualization, Data curation, Funding acquisition, Investigation, Methodology, Project administration, Writing—review and editing. SS: Methodology, Writing—review and editing. NS: Investigation, Project administration, Resources, Writing—review and editing. AK: Project administration, Visualization, Writing—review and editing. JP: Conceptualization, Supervision, Writing—review and editing. TF: Conceptualization, Investigation, Resources, Supervision, Writing—review and editing. JM: Conceptualization, Resources, Supervision, Writing—review and editing. WW: Conceptualization, Funding acquisition, Resources, Validation, Writing—review and editing. WH: Conceptualization, Supervision, Validation, Writing—review and editing. CG: Conceptualization, Funding acquisition, Methodology, Resources, Supervision, Validation, Writing—original draft.

## References

[1] Kyu HH Abate D Abate KH Abay SM Abbafati C Abbas N. Global, regional, and national disability-adjusted life-years (DALYs) for 359 diseases and injuries and healthy life expectancy (HALE) for 195 countries and territories, 1990–2017: a systematic analysis for the global burden of disease study (2017). The Lancet. (2018) 392:1859-1922. 10.1016/s0140-6736(18)32335-3 30415748 PMC6252083

[2] Pandian JD Kalkonde Y Sebastian IA Felix C Urimubenshi G Bosch J. Stroke systems of care in low-income and middle-income countries: challenges and opportunities. Lancet. (2020). 396:1443-1451. 10.1016/S0140-6736(20)31374-X 33129395

[3] Owolabi MO Thrift AG Martins S Johnson W Pandian J Abd-Allah F. The state of stroke services across the globe: report of world stroke organization-world health organization surveys. Int J Stroke. (2021) 16:889–901. 10.1177/17474930211019568 33988062 PMC8800855

[4] Bank W. World bank country and lending groups. In:Group WB (ed.) (2023).

[5] Thapa L Shrestha S Kandu R Ghimire MR Ghimire S Chaudhary NK. prevalence of stroke and stroke risk factors in a south-western community of nepal. J Stroke Cerebrovascul Dis Off J Nat Stroke Assoc. (2021). 30:105716. 10.1016/j.jstrokecerebrovasdis.2021.105716 33725500

[6] Thapa l. A plea from nepal. Int J Stroke. (2011). 6:98. 10.1111/j.1747-4949.2011.00589.x 21371268

[7] Thapa L Shrestha S Shrestha P Bhattarai S Gongal DN Devkota UP. Feasibility and efficacy of thrombolysis in acute ischemic stroke: a study from national institute of neurological and allied sciences, kathmandu, nepal. J Neurosci Rural Pract. (2016). 7:55–60. %2 26933345. 10.4103/0976-3147.172161 26933345 PMC4750341

[8] Thapa L Sharma N Poudel RS Bhandari TR Bhagat R Shrestha A. Knowledge, attitude, and practice of stroke among high school students in Nepal. J Neurosci Rural Pract. (2016) 7:504–9. 10.4103/0976-3147.188635 27695228 PMC5006460

[9] Nepal G Yadav JK Basnet B Shrestha TM Kharel G Ojha R. Status of prehospital delay and intravenous thrombolysis in the management of acute ischemic stroke in Nepal. BMC Neurol. (2019) 19:155. 10.1186/s12883-019-1378-3 31288770 PMC6615236

[10] Phuyal S Poudel R Shrestha GS Dawadi K Rauniyar VK Thapa L. Endovascular management of acute ischaemic stroke in Nepal. Lancet Glob Health. (2020) 8: e635-e636. 10.1016/S2214-109X(20)30071-1 32353306

[11] Chandra A Rajbhandari P Pant B. Acute stroke management: the plight of Nepal. Neurology. (2019). 92:1022–3. 10.1212/WNL.0000000000007528 31110146

[12] Paudel R. Stroke epidemiology and outcomes of stroke patients in Nepal: a systematic review and meta-analyis. [Manuscript submitted for publication]. (2023). 10.1186/s12883-023-03382-5 37749496 PMC10519080

[13] Caso V Martins S Mikulik R Middleton S Groppa S Pandian JD. Six years of the angels initiative: aims, achievements and future directions to improve stroke care worldwide. Int J Stroke 2023:17474930231180067. 10.1177/17474930231180067 37226325 PMC10507995

[14] Lindsay P Furie KL Davis SM Donnan GA Norrving B. World stroke organization global stroke services guidelines and action plan. Int J Stroke: Offic J Int Stroke Soc. (2014) 100:4–13. %2 25250836. 10.1111/ijs.12371 25250836

[15] Mikulík R Caso V Bornstein NM Svobodová V Pezzella FR Grecu A. Enhancing and accelerating stroke treatment in eastern european region: methods and achievement of the ESO EAST program. Eur Stroke J. (2020). 5:204-212. 10.1177/2396987319897156 32637654 PMC7313365

[16] Tunkl C Paudel R Thapa L Tunkl P Jalan P Chandra A. Are digital social media campaigns the key to raise stroke awareness in low-and middle-income countries? a study of feasibility and cost-effectiveness in nepal. [Manuscript submitted for publication]. (2023). 10.1371/journal.pone.0291392 37682967 PMC10490866

[17] Yeghiazaryan N Isahakyan A Zubalova L Hovhannisyan Y Sahakyan G Chekijian S. Stroke care in Armenia: recent developments. Euro Stroke J. (2023) 8:28–34. 10.1177/23969873221108739 36793742 PMC9923127

[18] Tiu C Terecoasă EO Tuṭǎ S Bǎlaṣa R Simu M Sabău M. Quality of acute stroke care in romania: achievements and gaps between 2017 and (2022). Euro Stroke J. (2023) 8:44–51. 10.1177/23969873221108746 36793744 PMC9923129

[19] Gdovinova Z Kovačik M Urbani D. How stroke care has changed in slovakia in the last 5 years. Eur Stroke J. (2023). 8:52–58. 10.1177/23969873221115457 36793747 PMC9923130

[20] Martins SCO Pontes-Neto OM Alves CV de Freitas GR Filho JO Tosta ED. Past, present, and future of stroke in middle-income countries: the Brazilian experience. Int J Stroke: offic J Int Stroke Soc. (2013) 100:106–111. 10.1111/ijs.12062 23692595

[21] John L William A Dawar D Khatter H Singh P Andrias A. Implementation of a physician-based stroke unit in a remote hospital of north-east india-tezpur model. J Neurosci Rural Pract. (2021). 12:356–361. 10.1055/s-0041-1723099 33927525 PMC8064833

[22] Pandian JD Joy SA Justin M Premkumar AJ John J George AD. Impact of stroke unit care: an indian perspective. Int J Stroke: Offic J Int Stroke Soc. (2011) 6:372–3. 10.1111/j.1747-4949.2011.00626.x 21745352

[23] Topcuoglu MA Ozdemir AO. Acute stroke management in turkey: current situation and future projection. Eur Stroke J. (2023) 8:16–20. 10.1177/23969873221103943 36793740 PMC9923125

[24] Roushdy T Aref H Kesraoui S Temgoua M Nono KP Gebrewold MA. Stroke services in Africa: what is there and what is needed. Int J Stroke. (2022) 17:972–82. 10.1177/17474930211066416 35034522

