# Supplementary material for: Implementing stroke care in a lower-middle-income country: results and recommendations based on an implementation study within the Nepal Stroke Project
Source: Front Neurol. 2023 Oct 24;14:1272076. doi: 10.3389/fneur.2023.1272076 (PMC10628475; doi:10.3389/fneur.2023.1272076)
Supplement: Supplementary file 1 [file Data_Sheet_1.PDF]

Year	Month	Quality Improvement	Stroke Awareness	Quality Monitoring	Stroke Care Policy
2020	Jul				Contacting Nepal Stroke Association (NSA)<br>Project Outline
	Aug				Gathering information on funding
	Sep				Meeting with NSA
	Oct				Submission GIZ Funding Proposal
	Nov	Angels Train the Trainer (TtT) Workshop			
	Dec				Systematic Research Study Proposal
2021	Mar				Kick-Off-Meeting<br>NHRC Ethical clearance proposal
	Jun				Acceptance GIZ Funding
	Jul				Meeting with CDC Ludhiana
	Sep	TtT Workshop			
	Oct	CME Kathmandu and Bairahawa	World Stroke Day Walkathon Kathmandu		
	Nov		NSP Website finalized		
	Dec	TtT Workshop	Cooperation with WSO		
2022	Jan	Design treatment protocols<br>Workshop CMC Chitwan	Start Social Media Campaign	Hospital visits Kathmandu	
	Feb	Workshop Pokhara, Manipal & Nepalganj	Facebook organic and paid started		
	Mar	Workshops in Pokhara (WRH), BPKIHS,<br>Koshi Hospital & Lumbini		Assessment Province No 1	Project Scetch EKFS<br>Meeting WHO & MOHP<br>National Road Map creation<br>Meetings Angels & WSO
	Apr	Workshop Janakpur	Health Camp Janakpur<br>Start TikTok Account		
	May	CME Kathmandu<br>Stroke Care Masterclass Manipal			Stroke Team BPKIHS established
	Jun	CME Narayanghat		Assessment Karnali	Meeting Global Health Institute
	Jul	TtT Workshop		Quality Monitoring Session	EKFS Application<br>GIZ Zwischenbericht<br>GIZ Meeting & Vertragsänderung
	Aug	CME Dharan<br>Stroke Guideline Nepal<br>Training Manual Nepal			Meeting WHO and MOHP
	Sep	Meeting Telecare providers<br>Online Series Vol 1 & 2	Flyer Distribution BHOJ started		
	Oct	Online Series Vol 3 & 4<br>Start Nurseletter<br>World Stroke Congress	World Stroke Day Dharan<br>Flyer Distribution KTM		
	Nov	Online Series Vol 5, 6 & 7<br>Workshop Chitwan<br>Neurointerventionalist Meeting		Paper Public Awareness	Submission GIZ Follow up Proposal<br>Project Outline NSP Phase II<br>Meeting WHO and MOHP
	Dec	Online Series Vol 8 & 9<br>Workshops BPKIHS Nurses & Manipal			
2023	Jan	Online Series Vol 10	BHOJ Resto Flex Distribution<br>Facebook Intensive Campaign<br>WSO Blog Article Public Awareness		Online Meeting WHO and MOHP
	Feb	Online Series Vol 11<br>Meeting Mechi Zonal Hospital, Koshi Hospital & BPKIHS<br>Workshop Koshi Hospital<br>Stroke Care Symposium Kathmandu<br>Design Trainingmanuals	Presentation Website GIZ	RES-Q Workshop	Meeting Manipal Hospital Board<br>Teleneurological Assessement Province No 1<br>Meeting WHO & MOHP<br>Meeting GIZ
	Mar	Online Series Vol 12<br>Meeting Dhulikel<br>Workshop WRH & Nepalganj<br>Workshop Manipal Nurses<br>Angels TtT Workshop	GIZ Blog Artikel Symposium		Meeting German Embassy<br>Presentation NSP at WSO
	Apr	Online Series Vol 13		Submission Ethical Clearance Qualitative Study	Abstract submission WSC
	May	Online Series Vol 14<br>Start weekly case series<br>Assessment call Nepalganj Bheri	Digital billboards displayed		
